# The impact of self-reported sensory impairment on cognitive function using the Korean longitudinal study of aging survey data

**DOI:** 10.1038/s41598-022-22840-7

**Published:** 2022-10-25

**Authors:** Hye Jin Joo, Jae Hong Joo, Seung Hoon Kim, Junhyun Kwon, Eun-Cheol Park

**Affiliations:** 1 Department of Public Health, Graduate School, Yonsei University, Seoul, Republic of Korea; 2 Institute of Health Services Research, Yonsei University, Seoul, Republic of Korea; 3 Department of Preventive Medicine and Institute of Health Services Research, Yonsei University College of Medicine, 50 Yonsei-ro, Seodaemun-gu, Seoul, 03722 Republic of Korea

**Keywords:** Diseases, Health care, Neurology, Risk factors, Signs and symptoms

## Abstract

Recent studies suggest that sensory impairment is related to cognitive function at older ages. Therefore, we aimed to investigate the impact of sensory impairment on cognitive function in the Korean population. We used the Korean Longitudinal Study of Aging data from 2006 to 2018. Cognitive function was measured by the Korean version of the Mini-Mental State Examination scale. A score < 24 at the time of assessment was defined as cognitive impairment. Sensory impairment was assessed according to the self-reported levels of hearing or vision, and the development of sensory impairment was investigated using records of prior survey. We used the generalized estimating equation model to determine association between cognitive function and sensory impairment. A total of 4844 participants (age range: 47–95 years; mean age: 58) were included in the study. Compared to people without sensory impairment, people with a single sensory impairment of hearing or vision had a higher risk of cognitive impairment (odds ratio (OR) = 1.65 [95% confidence interval (CI), 1.49–1.82]). People with dual sensory impairment had the greatest risk of cognitive impairment (OR = 3.23 [95% CI, 2.52–4.12]). The findings suggested the need for timely assessment of sensory function in older persons, which may be useful in identifying individuals at risk for cognitive impairment.

## Introduction

The prevalence of hearing or visual impairment, and dual sensory impairment (DSI) increases with advancing age. The impairment of sensory function that affects health-related problems in older adults may be the result of aging, but may be the result of accumulation of genetic, environmental, and lifestyle factors^1,2^ Of the Americans aged 70 years and over, 33% are affected by hearing loss and 18% by vision impairment^3^. Dual sensory impairment, defined as a combination of hearing and vision loss, has a prevalence of about 5%–21%^4^. Sensory impairment is considered a chronic condition in an aging society^5,6^. Hearing and visual impairments are likely to be overlooked compared to the burden of other health conditions^7^. However, the quality of life for people with sensory impairment may be lower than for people without^5,8,9^.

Sensory impairments are not only related to functional problems in older adults, but also affect mental health conditions^10,11^. Sensory impairment is associated with an increased mortality rate and an increased risk of physical and social functional difficulties^12^. Additionally, several studies have shown that hearing and vision impairments in older adults increase the risk of cognitive dysfunction and dementia^13–15^.

Cognitive impairment often occurs with sensory impairment in older adults^16,17^. While cognitive function may decline with age, it may also indicate the onset of geriatric neurodegenerative diseases. Cognitive impairment has adverse health consequences, including diminished health-related quality of life^18^, frailty^19^, and high mortality risk^20^. Therefore, screening to prevent cognitive impairment is important for the mental health and successful aging of older adults.

There are various pathways by which sensory impairments affect cognitive function. Sensory impairment could isolate individuals from society, which can affect cognitive function by causing a decrease in cognitive stimulation. In addition, changes in hearing and vision that decrease with age are mostly peripheral and can affect central processing^21^. A prospective cohort study suggests that central auditory dysfunction may be used as an early risk marker for dementia^22^. Another study has also found that visual impairment is associated with an increased risk and severity of Alzheimer’s disease^23^. As such, cognitive impairment may be modifiable and preventable rather than an inevitable health outcome.

Most previous studies have focused on single sensory disorders, and there is a paucity of evidence on the effects of dual sensory disorders on cognitive function when compared with single sensory impairment^14,24,25^. When one sensory function impaired, the other sensory function may offset for it. However, in older age, both hearing and vision are likely to decrease, which may limit this offset^21^. Moreover, there is a lack of research on the relationship between sensory disorders and cognitive function among Koreans.

We hypothesized that onset of single or dual sensory impairment would be associated with cognitive impairment. Therefore, this study aimed to investigate the effects of sensory impairment on cognitive function among middle-aged and older Koreans, using a population-based longitudinal data.

## Methods

### Data collection and study population

We used data from the first (2006) to the seventh (2018) waves of the Korea Longitudinal Study of Aging (KLoSA). The KLoSA is a prospective population-based study designed to investigate the factors related to aging in community-dwelling Korean adults, including demographics, family and social networks, physical and mental health status, employment and retirement, income, and wealth. The surveyed participants were aged 45 years or older and were selected by multistage stratified probability sampling method. The study was initiated by the Korea labor institute in 2006, and the sample has been followed up at 2-yearly intervals for a total of seven waves. A more detailed description of the KLoSA can be found on the Korea Employment Information Service (KEIS) website (http://survey.keis.or.kr).

In 2006, the original panel sample was composed of 10,254 adults aged 45 years and over (born in 1961 or earlier) who resided in South Korea. We excluded those with cognitive impairment (n = 2686) from the first wave. In order to target only those who have newly developed sensory impairment, people with sensory impairment at baseline (n = 1391) were excluded from the study. We also excluded participants who contained missing values for all the variables from first wave to seventh wave (n = 2004). The total number of participants was 4844 in the final sample at the baseline (2008). Only subjects who participated from the first wave were used, and new participants during follow-up were not considered. The sample size for each wave is as follows; 2008 = 4844, 2010 = 4300, 2012 = 4181, 2014 = 4293, 2016 = 3954, 2018 = 3700.

### Cognitive function

The main objective of this study was to analyze the association of sensory impairment on cognitive function. Cognitive function was measured by Korean version of the Mini-Mental State Examination (K-MMSE) scale^26,27^. The K-MMSE comprised 19 questions in five cognitive function areas (orientation in time and place, registration, attention and calculation, memory recall, and visual construction). The subscale scores for these areas were summed up to derive an overall K-MMSE score ranging from 0 to 30, with higher scores indicating better cognitive function. In this study, K-MMSE score < 24 at the time of assessment was defined as cognitive impairment. Participants were categorized into two groups of either cognitive impairment (K-MMSE score < 24) or normal cognition (K-MMSE score ≥ 24)^26^.

### Sensory impairment

The main exposure of interest was the onset of sensory impairment. The survey required the participants to report their perception of hearing and vision. Individuals who use hearing aids/glasses reported their hearing/vision with those aids. That is, hearing was investigated on the basis of hearing ability when using hearing aids in the case of hearing aid users, and vision was investigated on the basis of corrected vision. The level of self-reported hearing and vision were rated on a 5-point scale (excellent, very good, good, fair, or poor). We categorized the levels as ‘normal’ (excellent, very good, or good) or ‘poor’ (fair or poor). This type of self-reported evaluation and classification has also been used in previous longitudinal studies of aging^10,28^. Onset of sensory impairment was measured by changes in hearing or visual status in the previous and subsequent years. We applied a lag-time option across all 2-year units in the overall period to detect changes in sensory impairment compared to that of prior survey year. Therefore, the baseline for this study was 2008. Compared to the prior wave, the case where the sensory function changed from ‘normal’ to ‘poor’ was defined as the onset of sensory impairment. When one of the hearing or visual impairments developed, this was defined as single sensory impairment. When hearing and visual impairment occurred at the same time, this was defined as dual sensory impairment. Therefore, we categorized the respondents into three groups according to their development of sensory impairment over the two years, as follows: No → No, No → Single impairment, No → Dual sensory impairment. ‘No → No’ was set as a reference group. Also, for the additional analysis to analyze the association between sensory impairment types and cognitive impairment, the single impairment was stratified into hearing and visual impairment.

### Covariates

In this study, demographic, socioeconomic, and health-related characteristics were included as covariates in the fully adjusted models. The included covariates were age (45–54, 55–64, 65–74, ≥ 75 years); sex; educational level (middle school or below, high school, college or above); region (metropolitan, urban, rural); economic activity (active, inactive); equivalized household income (divide into quartiles); marital status (married, divorced, separated, or widowed, unmarried); the number of chronic disease (0, 1, ≥ 2; hypertension, diabetes, cancer or malignant tumors, chronic lung disease, liver disease, heart disease, cerebrovascular disease, psychiatric disease, arthritis and rheumatism); limitations in activities of daily living (ADL; disabled, normal); limitations in instrumental ADL (IADL; disabled, normal); body mass index (BMI; normal or underweight [BMI < 23]; overweight [23 ≤ BMI < 25], obese [BMI ≥ 25]); smoking status (current, past, never); alcohol consumption (current, past, never); regular exercise (yes, no); depressive symptom using CES-D 10 scores (yes, no); and year.

### Statistical analysis

Chi-square test was used to evaluate and compare the general characteristics of the study population. The statistical significance level was defined as a two-tailed *p* value of < 0.05. We also evaluated the relationship between sensory impairment and cognitive function using a generalized estimating equation (GEE) model that is useful for analyzing longitudinal data. The GEE model could account for time variations and correlations among repeated measurements observed in a longitudinal study^29^. Odds ratios (ORs) and 95% confidence intervals (CIs) were calculated to compare parameters between those who had cognitive impairment and those with normal cognition. In addition, in this statistical model, multicollinearity was tested using the variance inflation factors that were all below the generally accepted threshold of 5–10, used to indicate collinearity^30^. All the statistical analyses were performed using the SAS 9.4 software (SAS Institute, Cary, NC, USA).

### Ethical standards

Ethical approval was not required as the Korean Longitudinal Study of Aging Data provides anonymous, secondary data that is publicly available for scientific use.

## Results

Table 1 shows the general characteristics of participants at baseline, which is the first point of change (2006 → 2008). The baseline for this study is 2008 as the lag-time option is applied to detect changes in sensory impairment compared to previous survey wave. A total of 4844 participants were included in the study. Their age range was 47 to 95 years old and the mean age was 58. Of which 11.4% (n = 551) and 88.6% (n = 4293) were classified with cognitive impairment and normal cognitive function, respectively. At baseline, out of 4844 participants, 16.3% (n = 788) had single sensory impairment such as hearing or vision, and 1.1% (n = 54) had dual sensory impairment. In 788 cases of single sensory impairment, 10.2% (n = 80) with hearing problems and 89.8% (n = 708) with visual problems. All covariates, such as demographic, socioeconomic, and health-related characteristics were significantly associated with cognitive function (all *p* values < 0.05).

**Table 1:** General characteristics of the study subjects at the first point of change (2006 → 2008).

Variables	Cognitive impairment (K-MMSE < 24)					*P* value
	Total (n = 4,844)	Yes (n = 551)		No (n = 4,293)		
	N	N	%	N	%	
**Sensory impairment**						< .0001
No → No	4002	381	9.5	3621	90.5	
No → Single	788	145	18.4	643	81.6	
No → Dual	54	25	53.7	29	46.3	
**Age**						< .0001
45–54	1741	65	3.7	1676	96.3	
55–64	1614	133	8.2	1481	91.8	
65–74	1153	229	19.9	924	80.1	
≥ 75	336	124	36.9	212	63.1	
**Sex**						< .0001
Male	2379	197	8.3	2182	91.7	
Female	2465	354	14.4	2111	85.6	
**Educational level**						< .0001
Middle school or below	2461	452	18.4	2009	81.6	
Hight school	1730	81	4.7	1649	95.3	
College or above	653	18	2.8	635	97.2	
**Region**						< .0001
Metropolitan area	2177	190	8.7	1987	91.3	
Urban area	1616	201	12.4	1415	87.6	
Rural area	1051	160	15.2	891	84.8	
**Economic activity**						< .0001
Active	2504	162	6.5	2342	93.5	
Inactive	2340	389	16.6	1951	83.4	
**Income**						< .0001
Low (quartile 1)	1036	230	22.2	806	77.8	
Middle-low (quartile 2)	1218	158	13.0	1060	87.0	
Middle-high (quartile 3)	1261	95	7.5	1166	92.5	
High (quartile 4)	1329	68	5.1	1261	94.9	
**Marital status**						< .0001
Married	4162	405	9.7	3757	90.3	
Separated, divorced, widow, single	682	146	21.4	536	78.6	
**Chronic disease**^**†**^						< .0001
0	2625	196	7.5	2429	92.5	
1	1405	190	13.5	1215	86.5	
≥ 2	814	165	20.3	649	79.7	
**ADL**						< .0001
Disabled	46	21	45.7	25	54.3	
Not disabled	4798	530	11.0	4268	89.0	
**IADL**						0.0036
Disabled	310	51	16.5	259	83.5	
Not disabled	4534	500	11.0	4034	89.0	
**BMI (kg/m**^**2**^**)**						0.0002
Normal or underweight (BMI < 23)	2213	267	12.1	1946	87.9	
Overweight (23 ≤ BMI < 25)	1542	135	8.8	1407	91.2	
Obese (BMI ≥ 25)	1089	149	13.7	940	86.3	
**Smoking status**						< .0001
Current smoker	990	69	7.0	921	93.0	
Ex-smoker	611	51	8.3	560	91.7	
Never	3243	431	13.3	2812	86.7	
**Alcohol consumption**						< .0001
Current drinker	2062	143	6.9	1919	93.1	
Ex-drinker	392	51	13.0	341	87.0	
Never	2390	357	14.9	2033	85.1	
**Regular exercise**						< .0001
Yes	2008	137	6.8	1871	93.2	
No	2836	414	14.6	2422	85.4	
**Depressive symptoms**						< .0001
Yes	1870	363	19.4	1507	80.6	
No	2974	188	6.3	2786	93.7	
**Total**	**4844**	**551**	**11.4**	**4293**	**88.6**	

*ADL* activities of daily living; *IADL* instrumental activities of daily living; *BMI* body mass index; *MMSE* mini-mental state examination.

^†^Hypertension, diabetes, cancer or malignant tumors, chronic lung disease, liver disease, heart disease, cerebrovascular disease, psychiatric disease, arthritis, and rheumatism.

Table 2 presents the results of the GEE model for the impact of sensory impairment on cognitive function that were repeatedly measured in the two-yearly units from 2006 to 2018 as ORs. All analyses were performed after adjusting for covariates. The “No → No” group that maintained normal sensory function, was the reference group. The ORs for cognitive impairment was 1.65 times higher in the single sensory impairment group than in the reference group (OR = 1.65 [95% CI, 1.49–1.82]). The ORs for cognitive impairment was highest in the DSI group, where hearing and visual impairment occurred simultaneously (OR = 3.23 [95% CI, 2.52–4.12]). The results did not change even when a sensitivity analysis was performed on the entire sample, including all missing values.

**Table 2:** Results of generalized estimate equation model on cognitive function according to sensory impairment.

Variables	Cognitive impairment	
	OR	95% CI
**Sensory impairment**		
No → No	1.00	
No → Single	1.65	(1.49–1.82)
No → Dual	3.23	(2.52–4.12)
**Age**		
45–54	1.00	
55–64	1.20	(1.01–1.43)
65–74	2.34	(1.93–2.84)
≥ 75	3.89	(3.12–4.84)
**Sex**		
Male	1.00	
Female	1.31	(1.11–1.53)
**Educational level**		
Middle school or below	2.78	(2.25–3.44)
Hight school	1.45	(1.16–1.81)
College or above	1.00	
**Region**		
Metropolitan area	1.00	
Urban area	1.24	(1.10–1.39)
Rural area	1.44	(1.28–1.63)
**Economic activity**		
Active	1.00	
Inactive	1.55	(1.39–1.72)
**Income**		
Low (quartile 1)	1.29	(1.11–1.49)
Middle-low (quartile 2)	1.11	(0.96–1.28)
Middle-high (quartile 3)	1.00	(0.87–1.15)
High (quartile 4)	1.00	
**Marital status**		
Married	1.00	
Separated, divorced, widow, single	1.15	(1.02–1.29)
**Chronic disease**^**†**^		
0	1.00	
1	1.03	(0.91–1.17)
≥ 2	1.13	(1.00–1.29)
**ADL**		
Disabled	2.47	(1.80–3.37)
Not disabled	1.00	
**IADL**		
Disabled	1.49	(1.27–1.76)
Not disabled	1.00	
**BMI (kg/m**^**2**^**)**		
Normal or underweight (BMI < 23)	1.00	
Overweight (23 ≤ BMI < 25)	0.85	(0.77–0.94)
Obese (BMI ≥ 25)	0.90	(0.80–1.01)
**Smoking status**		
Current smoker	0.94	(0.78–1.13)
Ex-smoker	1.01	(0.86–1.20)
Never	1.00	
**Alcohol consumption**		
Current drinker	1.00	(0.88–1.14)
Ex-drinker	1.08	(0.93–1.25)
Never	1.00	
**Regular exercise**		
Yes	1.00	
No	1.51	(1.37–1.66)
**Depressive symptoms**		
Yes	1.78	(1.64–1.94)
No	1.00	
**Year**		
2008	1.00	
2010	1.14	(1.01–1.29)
2012	0.91	(0.80–1.04)
2014	1.21	(1.07–1.38)
2016	1.07	(0.93–1.22)
2018	1.13	(0.97–1.31)

*ADL* activities of daily living; *IADL* instrumental activities of daily living; *BMI* body mass index; *MMSE* mini-mental state examination; *CI* confidence interval; *OR* odds ratio.

^†^Hypertension, diabetes, cancer or malignant tumors, chronic lung disease, liver disease, heart disease, cerebrovascular disease, psychiatric disease, arthritis, and rheumatism.

**Figure 1:**
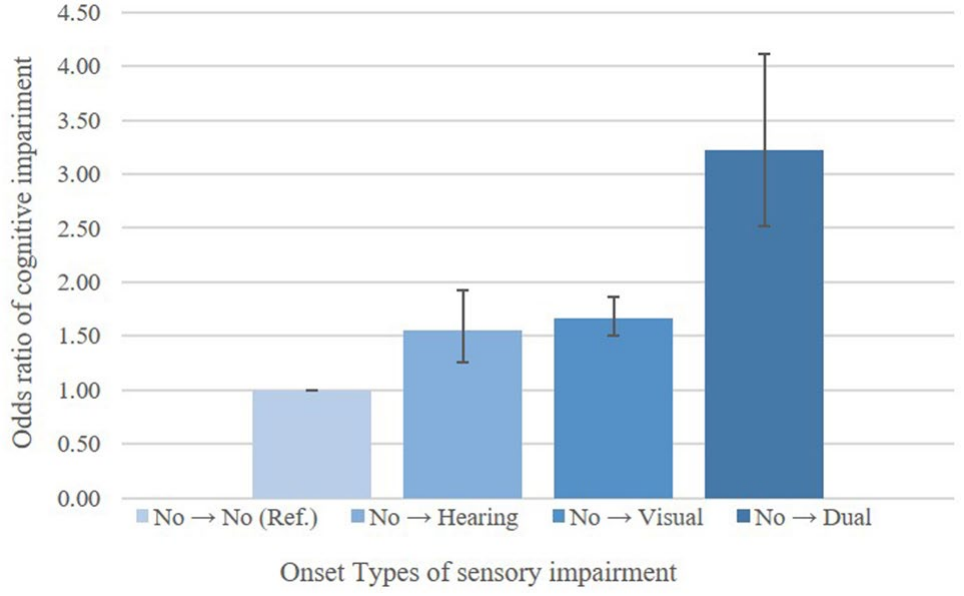
Results of generalized estimating equation model on cognitive function according to sensory impairment by types. Analysis was adjusted for all demographic, socioeconomic, and health-related factors considered in the study. Error bars represent 95% confidence intervals.

In order to analyze the association between the onset of sensory impairment by types and cognitive impairment, all the independent variables were adjusted for, and GEE analysis was performed (Fig. 1, Supplementary Table S1). We observed that the odds of cognitive impairment were 1.55 times and 1.67 times higher, respectively, when hearing impairment and visual impairment occurred newly (OR = 1.55 [95% CI, 1.25–1.92] for hearing; 1.67 [95% CI, 1.50–1.86] for visual). The values were statistically significant.

## Discussion

In this study, we examined the impact of changes in sensory impairment on cognitive function in Koreans aged 45 years or older using the KLoSA data. We found that those who reported the development of sensory impairment were more likely to experience cognitive impairment than older adults without these impairments. Moreover, older adults with dual sensory impairment had a higher risk of cognitive impairment than those with single sensory impairment such as hearing or vision.

Several previous studies also found a relationship between sensory impairment and cognitive functions. A study found that individuals with hearing loss have accelerated cognitive decline of 30–40% and increased risk of incident cognitive impairment of 24% compared to individuals with normal hearing^25^. One longitudinal study examined 253 individuals aged 45–64 years at baseline and found that hearing impairment was associated with faster cognitive decline over 20 years^31^. As a result of meta-analysis of 36 studies, age-related hearing loss was significantly associated with multi-domain cognitive decline, cognitive impairment, and dementia acceleration^32^. One community-based prospective study^33^ found visual impairment to be predictive of subsequent functional impairment in older persons, while other studies showed a decline in cognition and functional activities^34–36^. A meta-analysis of 40 studies found that people with visual impairments were associated with an approximately 2-fold odds of prevalent or incident cognitive impairment^37^.

Our results on changes in sensory impairment types showed that those with dual sensory impairment had higher risk of cognitive impairment, which is in line with several previous studies. According to a prospective cohort study, dual sensory impairment was associated with 86% increased risk for all‐cause dementia and a 112% increased risk for Alzheime’s disease compared with having no sensory impairment^14^. A study of women aged 69 years and over reported that dual sensory impairment increased the risk of cognitive decline twofold compared to no impairment over a 4-year period^38^. Another study reported that those with dual sensory impairment had approximately twofold increased odds of cognitive decline, even after adjusting for other factors such as age, medical comorbidities, smoking, and walking speed^38^. A study of older Australians also found a higher rate of cognitive impairment among those with dual sensory impairment compared to those without^24^. Similar results have been reported in Iceland showing approximately 30% higher rate of cognitive impairment among older adults with dual sensory impairment^39^. A longitudinal study in China also found that cognitive function declined even more with dual sensory impairment^13^.

Sensory impairment may lead to cognitive impairment by cumulative effect of decreased sensory stimulation. This has causal effects on sensory loss, social isolation, depression, and decreased physical activity. Impaired communication due to sensory impairment limits participation in social activities and increase the difficulty of maintaining social networks^40^. In addition, protracted lack of sense can reduce the chances of intellectually stimulating exchanges, eventually reducing the general level of cognitive ability^41^. Sensory impairment and cognitive impairment could be caused by common pathological processes such as vascular disease. For example, β-amyloid pathology, pathological features of Alzheimer’s disease, could damage both sensory and cognitive abilities. Sensory disorders can increase cognitive load, limiting neural resources required for optimal performance of cognitive tasks, and loss of senses can affect changes in brain structure and function^42^.

The limitations of this study should be considered when interpreting our results. First, the information for sensory function data used in this study were self-reported, so there may lead to biases in the respondents’ responses. Specifically, we used self-reported measures of hearing and visual functions. Although self-reported assessments of hearing or vision have been widely used in population-based surveys^10,13,28^, it may not represent the subject’s health condition as is, whether intended or not. Previous studies have shown that older adults participants overestimate the adequacy of their vision when asked for a self‐evaluation^43^, and underestimate their hearing impairment^44^. Another study found that older adults tend to overestimate their hearing and report fewer hearing problems^45^. Therefore, studies should seek to replicate our findings using objective measures of hearing and vision. Second, only MMSE was used as a screening indicator for cognitive impairment. In the KLoSA data, MMSE was the only indicator that could check cognitive function. Further studies need to use clinical tools to assess cognitive function. Third, we could not measure the cause of sensory impairment in the participants due to data limitations. To minimize this limitation, we adjusted for variables that could affect the occurrence of sensory impairments or changes in sensory function state (e.g., chronic disease). It is also necessary to consider missing data due to non-response. Participants who recovered from sensory impairment were also not considered. Finally, this study used an observational study design, which prevented us from directly drawing causal inference. Although we adjusted for numerous potential confounders, some residual confounding may still persist. Despite these limitations, this study had a relatively large sample size, is representative of the community-dwelling Korean population aged 45 years and over, and documented an understudied association.

## Conclusion

This study highlighted that developing sensory impairment was related with cognitive function in middle-aged and older adults in South Korea. Specifically, the findings of our study provide further evidence implying a greater relationship between dual sensory impairment and cognitive function. Considering Korea’s rapidly aging population, this is a salient topic with important public health implications. Timely assessment of sensory function in older persons may be useful in identifying individuals at risk of cognitive impairment. Our research findings could provide health policy makers and professionals with valuable information about the development of intervention strategies to minimize negative consequences for cognitive ability by preventing sensory loss or optimal treatment of sensory impairment.

## Supplementary Information

Supplementary Information 1.

## Data Availability

The Korea Employment Information Service (KEIS) website (http://survey.keis.or.kr).
