# Supplementary material for: The impact of self-reported sensory impairment on cognitive function using the Korean longitudinal study of aging survey data
Source: Sci Rep. 2022 Oct 25;12:17907. doi: 10.1038/s41598-022-22840-7 (PMC9596449; doi:10.1038/s41598-022-22840-7)
Supplement: Supplementary file 1 — Supplementary Information 1. [file 41598_2022_22840_MOESM1_ESM.docx]

**The Impact of Self-reported Sensory Impairment on Cognitive Function Using the Korean Longitudinal Study of Aging Survey Data**

Hye Jin Joo ^1,2^: hjjoo22@yuhs.ac

Jae Hong Joo ^1,2^: jhj3040@yuhs.ac

Junhyun Kwon ^1,2^: judekwon@yuhs.ac

Seung Hoon Kim^2,3^: shoonkim@yuhs.ac

Eun-Cheol Park ^2,3^*: ecpark@yuhs.ac

^1^Department of Public Health, Graduate School, Yonsei University, Seoul, Republic of Korea

^2^Institute of Health Services Research, Yonsei University, Seoul, Republic of Korea

^3^Department of Preventive Medicine, Yonsei University College of Medicine, Seoul, Republic of Korea

***Corresponding author:** **Eun-Cheol Park, MD, PhD**

Department of Preventive Medicine and Institute of Health Services Research, Yonsei University College of Medicine

50 Yonsei-ro, Seodaemun-gu, Seoul 03722, Republic of Korea

Tel: +82-2-2228-1862; E-mail: ecpark@yuhs.ac; Fax: +82-2-392-8133

**Supplementary**

| **Supplementary Table S1. Results of generalized estimating equation model on cognitive function according to sensory impairment by types** | | | | |
| --- | --- | --- | --- | --- |
| **Variables** | **Cognitive impairment** | | | |
|  | **OR^*^** | **95% CI** | | |
| **Onset by type of sensory impairment** |  |  |  |  |
| No → No (n=4,721) | 1.00 |  |  |  |
| No → Hearing (n=117) | 1.55 | (1.25 | - | 1.92) |
| No → Visual (n=966) | 1.67 | (1.50 | - | 1.86) |
| No → Dual (n=111) | 3.22 | (2.52 | - | 4.12) |
| ^*^OR adjusted for age, sex, educational level, region, economic activity, income, marital status, chronic disease, Activities of Daily Living, Instrumental Activities of Daily Living, body mass index, smoking status, alcohol consumption, regular exercise, depressive symptoms. | | | | |
